# Revising the WHO verbal autopsy instrument to facilitate routine cause-of-death monitoring

**DOI:** 10.3402/gha.v6i0.21518

**Published:** 2013-09-13

**Authors:** Jordana Leitao, Daniel Chandramohan, Peter Byass, Robert Jakob, Kanitta Bundhamcharoen, Chanpen Choprapawon, Don de Savigny, Edward Fottrell, Elizabeth França, Frederik Frøen, Gihan Gewaifel, Abraham Hodgson, Sennen Hounton, Kathleen Kahn, Anand Krishnan, Vishwajeet Kumar, Honorati Masanja, Erin Nichols, Francis Notzon, Mohammad Hafiz Rasooly, Osman Sankoh, Paul Spiegel, Carla AbouZahr, Marc Amexo, Derege Kebede, William Soumbey Alley, Fatima Marinho, Mohamed Ali, Enrique Loyola, Jyotsna Chikersal, Jun Gao, Giuseppe Annunziata, Rajiv Bahl, Kidist Bartolomeus, Ties Boerma, Bedirhan Ustun, Doris Chou, Lulu Muhe, Matthews Mathai

**Affiliations:** 1 Disease Control and Vector Biology, London School of Hygiene and Tropical Medicine, London, UK; 2 WHO Collaborating Centre for Verbal Autopsy, Division of Epidemiology and Global Health, Department of Public Health and Clinical Medicine, Umeå University, Umeå, Sweden; 3 Health Information and Statistics, WHO, Geneva, Switzerland; 4 International Health Policy Program, Thailand Ministry of Public Health, Nonthaburi, Thailand; 5 Health Policy and Strategic Bureau, Ministry of Public Health, Nonthaburi, Thailand; 6 Public Health and Health Systems, Swiss Tropical and Public Health Institute, Basel, Switzerland; 7 UCL Centre for International Health and Development, Institute of Child Health, London, UK; 8 Epidemiology and Health Evaluation Faculty of Medicine, Federal University of Minas Gerais, Minas Gerais, Brazil; 9 Genes and Environment Division of Epidemiology, Norwegian Institute of Public Health, Oslo, Norway; 10 Faculty of Medicine, University of Alexandria, Alexandria, Egypt; 11 Health Research and Development Division, Ghana Health Serfice, Accra, Ghana; 12 Headquarter, United Nations Population Fund (UNFPA), New York, USA; 13 MRC/Wits Rural Public Health and Health Transitions Research Unit (Agincourt), School of Public Health, Faculty of Health Sciences, University of the Witwatersrand, Johannesburg, South Africa; 14 Centre for Community Medicine, All India Institute of Medical Sciences, New Delhi, India; 15 Uttar Pradesh Center, Community Empowerment Lab, Uttar Pradesh, India; 16 Ifakara Health Institute, Dar es Salaam, Tanzania; 17 International Statistics Division, Centers for Disease Control and Prevention, Hyattsville, USA; 18 Afghan Public Health Institute, Afghanistan Ministry of Public Health, Kabul, Afghanistan; 19 INDEPTH Network Secretariat, INDEPTH Network, Accra, Ghana; 20 Public Health and HIV Section, The office of the United Nations High Commissioner for Refugees (UNHCR), Geneva, Switzerland; 21 Independent Consultant; 22 Monitoring of Vital Events, Health Metrics Network, Geneva, Switzerland; 23 Health Information and Analysis, Pan American Health Organization, Washington, DC, USA; 24 Division of Health Systems and Services Development, WHO Regional Office for the Eastern Mediterranean, Cairo, Egypt; 25 Health Information, Evidence and Research Policy, WHO Regional Office for Europe, Kobenhavn, Denmark; 26 Evidence-Based Health Situation and Trends Assessment, WHO Regional Office for the South Eastern Region, New Dehli, India; 27 Health Information, Evidence and Research Policy, WHO Regional Office for Western Pacific, Manila, Philipines; 28 Mediterranean Centre for Health Risk Reduction, WHO, Geneva, Switzerland; 29 Department of Child and Adolescent Health and Development, WHO, Geneva, Switzerland; 30 Department of Violence and Injury Prevention and Disability, WHO, Geneva, Switzerland; 31 Health Statistics and Informatics, WHO, Geneva, Switzerland; 32 Classification, Terminology and Standards Unit, WHO, Geneva, Switzerland; 33 Department of Reproductive Health and Research, WHO, Geneva, Switzerland; 34 Child and Adolescent Health and Development, WHO, Geneva, Switzerland; 35 Maternal, Newborn, Child and Adolescent Health, WHO, Geneva, Switzerland

**Keywords:** verbal autopsy, cause of death, vital registration, civil registration, vital statistics, World Health Organization, InterVA

## Abstract

**Objective:**

Verbal autopsy (VA) is a systematic approach for determining causes of death (CoD) in populations without routine medical certification. It has mainly been used in research contexts and involved relatively lengthy interviews. Our objective here is to describe the process used to shorten, simplify, and standardise the VA process to make it feasible for application on a larger scale such as in routine civil registration and vital statistics (CRVS) systems.

**Methods:**

A literature review of existing VA instruments was undertaken. The World Health Organization (WHO) then facilitated an international consultation process to review experiences with existing VA instruments, including those from WHO, the Demographic Evaluation of Populations and their Health in Developing Countries (INDEPTH) Network, InterVA, and the Population Health Metrics Research Consortium (PHMRC). In an expert meeting, consideration was given to formulating a workable VA CoD list [with mapping to the International Classification of Diseases and Related Health Problems, Tenth Revision (ICD-10) CoD] and to the viability and utility of existing VA interview questions, with a view to undertaking systematic simplification.

**Findings:**

A revised VA CoD list was compiled enabling mapping of all ICD-10 CoD onto 62 VA cause categories, chosen on the grounds of public health significance as well as potential for ascertainment from VA. A set of 221 indicators for inclusion in the revised VA instrument was developed on the basis of accumulated experience, with appropriate skip patterns for various population sub-groups. The duration of a VA interview was reduced by about 40% with this new approach.

**Conclusions:**

The revised VA instrument resulting from this consultation process is presented here as a means of making it available for widespread use and evaluation. It is envisaged that this will be used in conjunction with automated models for assigning CoD from VA data, rather than involving physicians.

Information on causes of death (CoD) is essential for planning, implementing, monitoring, and evaluating public health at all levels. However, death registration and CoD determination do not happen for many deaths occurring in low- and middle-income countries (LMICs), and the deaths of poorer people are much less likely to be recorded, compounding inequalities. Statistical modelling is used to fill the data gaps, for example, for maternal deaths and malaria mortality. Facilitating complete and accurate CoD determination and death registration in LMICs is therefore a high priority. In the medium-term, this will involve applying verbal autopsy (VA) not only in surveillance sites and household surveys but also as a routine part of civil registration and vital statistics (CRVS) systems (1, 2).

VA ascertains probable CoD through interviews carried out with caretakers of the deceased or witnesses of deaths. The method uses questionnaires to elicit pertinent information on signs, symptoms, and circumstances leading to death, generically described as indicators, which are subsequently interpreted into CoD. VA has been increasingly used in various contexts including disease surveillance, sample registration systems, outbreak investigation, and measuring the impact of public health interventions. Because vital registration coverage has not significantly improved in most LMICs, VA data collection has been conducted in a variety of settings such as clinical trials and large-scale epidemiological studies; demographic surveillance systems; national sample surveillance systems; and household surveys. The expanding use of VA in generating mortality data has led to a proliferation of different VA instruments (comprising a set of questions/indicators that elicit pertinent information on signs, symptoms and circumstances preceding death and a corresponding list of CoD) that has impaired data comparability across sites and over time. Limited attention has been given to standardization of CoD interpretation from VAs (3).

Users have different perspectives on the required level of accuracy and categories of cause-specific mortality data, with corresponding impacts on desirable characteristics of VA instruments (4). However, the need for regular nationally representative cause-specific mortality data in settings where a significant proportion of deaths are not medically certified can only be met by death registration including VA as part of national CRVS systems. This requires simpler VA instruments and operating procedures that can produce timely, readily usable and reliable cause-specific mortality data.

To produce a simplified VA instrument, the World Health Organization (WHO) carried out a systematic review of VA instruments and procedures, followed by an expert consultation. Based on accumulated experience from widely-used and validated VA procedures, consensus was reached on a simplified VA instrument for routine use in CRVS systems where deaths are not medically certified. The 2012 WHO VA instrument comprises a short CoD list aligned to the International Classification of Diseases and Related Health Problems, Tenth Revision (ICD-10) that is ascertainable from a limited number of indicators and amenable to automated processing. The design allows adding a narrative and locally relevant questions and diagnoses as needed. The rationale and processes used to develop the 2012 WHO VA instrument are presented in this article.

## VA instruments and procedures

The WHO first encouraged the use of lay reporting of health information in 1956, and from then through the 1990s, developed lay reporting forms and published key design features for studies based on VA methods. With the expanding diversity and use of VA instruments, demands for standardization led to the development of the WHO VA standards in 2007 that included (5):

1. VA questionnaires for three age groups (under 4 weeks; 4 weeks to 14 years; and 15 years and above);
2. CoD certification and coding resources consistent with ICD-10; and
3. A CoD list for VA prepared according to the ICD-10.

The 2007 WHO VA standards were partially based on a VA instrument developed by the London School of Hygiene and Tropical Medicine (LSHTM). The WHO standards expected that up to three physicians trained in VA coding would independently review individual questionnaire data – known as physician-certified VA (PCVA). This procedure has been used by the International Network for the Demographic Evaluation of Populations and their Health in Developing Countries (INDEPTH)^1^ and by the Sample Vital Registration with Verbal Autopsy (SAVVY).^2^

However, since PCVA is time-consuming and expensive, computerized coding of VA (CCVA) methods for interpreting VA data have been investigated. Validated CCVA methods can be algorithmic or probabilistic. Algorithmic methods follow a set of predefined diagnostic criteria that can be expert- or data-derived. The Tariff method is an additive algorithm that uses Tariff scores reflecting the importance and uniqueness of each symptom to each CoD. The Artificial Neural Network (ANN) method uses computer algorithms (machine learning), applying non-linear statistics to pattern recognition. The Random Forests method is a machine learning method for interpreting VA based on patterns of indicators from a ‘training dataset’ (6). Whereas algorithmic methods result in binary outcomes (yes or no) for a single CoD, probabilistic methods determine the probability of a range of multiple causes. The InterVA method applies Bayesian probabilistic methods to a matrix of indicators and CoD, using conditional probabilities derived from available data and expert opinion. This method has been available in the public domain since 2006 (7, 8). King and Lu's algorithmic method is able to estimate cause-specific mortality fractions (CSMFs) without individual CoD assignment. The Simplified Symptom Pattern (SSP) method is a data-driven Bayesian approach that combines the King and Lu and InterVA methods.

## Review of utilization of VA instruments and procedures

Despite attempts to standardize and harmonize VA instruments, there are multiple instruments in use (9–11). We conducted a systematic literature review to determine how VA instruments have been used and the uptake of the WHO VA standards published in 2007.

**Fig. 1:**
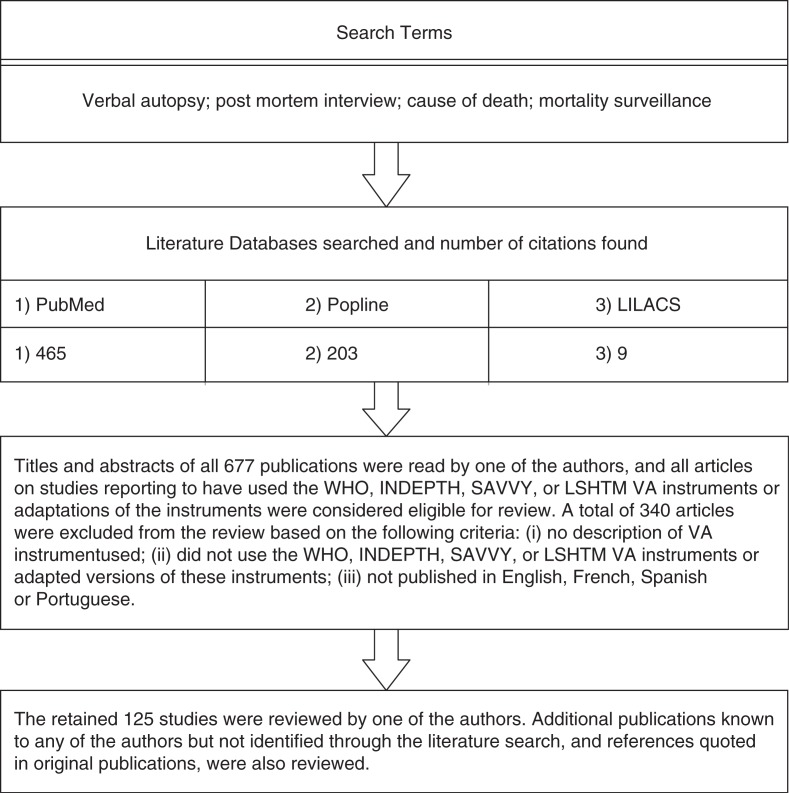
Illustration of literature search and review process.

The review included studies reported in peer-reviewed journals from 1986 up to early 2012. Figure 1 illustrates the review process. The WHO instruments and the three related ones briefly described above (INDEPTH, SAVVY and LSHTM) were included in the review. Instruments described as adapted from these were also included. Studies that did not provide details of the instrument used were excluded. A brief description of the 125 eligible studies is available as a Supplementary File.

Some studies applied different VA interpretation methods on the same dataset and were counted as a single study for the review of the use of the VA instruments. The selected VA instruments or their adaptations were reported to be used by 112 studies in 41 countries. Table 1 summarizes the identified studies, data collection period, and number of deaths certified, by each VA instrument. VA was mostly used as a research tool in longitudinal health and demographic surveillance and in intervention or epidemiological studies. The first study identified used an adapted version of an early WHO instrument to certify perinatal deaths in Nepal in 1989 (12). From the 112 reviewed studies, 104 reported the number of deaths certified, totalling 159,316. Studies using the INDEPTH instrument certified the largest number of deaths, ranging from 100 to 38,306 deaths with a mean of 4269.4, totalling 72,579 deaths (Table 1).

**Table 1:** Summary characteristics of reviewed studies (*n*=112) by type of VA instrument^a^

				% of studies with sites		
				
	Number of identified studies	Data collection period	Mean and range of number of deaths certified	Africa	Asia	Central and South America
WHO VA instrument	31 (27.7%)	1992–2010	620.1 (23–4 239)	61.3	32.3	16.1
Adapted WHO VA instrument	42 (37.5%)	1989–2010	1 347.5 (2–12 542)	35.7	59.5	7.1
INDEPTH VA instrument	17 (15.2%)	1996–2009	4 269.4 (100–38 306)	64.7	35.3	0
Adapted INDEPTH VA instrument	9 (8%)	1999–2010	590.7 (164–1 816)	100	0	0
SAVVY instrument	1 (0.9%)	2007–2010	145	100	0	0
Adapted SAVVY instrument	3 (2.7%)	2006–2008	258 (252–264)	33.3	0	66.7
LSHTM VA instrument	5 (4.5%)	1992–2002	407.3 (40–796)	80	20	0
Adapted LSHTM VA instrument	4 (3.6%)	2003–2007	2 304.3 (1 084–5 160)	25	75	0

a Percentages of studies conducted amount to more than 100% because some multicentre studies had sites in more than one continent.

VA has also been applied in national health surveys. In most surveys (e.g. Nepal Demographic and Health Survey (DHS) 2006, Ghana DHS 2008, Bangladesh DHS 2011), this involves the identification of deaths among children under 5 years in either the household schedule or the individual interview of women of reproductive age, followed by administration of a VA module. In Uganda, deaths among children under 5 identified in the DHS in 2007 were followed up in a subsequent survey in 2008. In the Afghanistan Mortality Survey 2010, a VA was administered for deaths of all ages. In Mozambique, a post census VA was conducted in 2008. All surveys ask for medical certification of the CoD, but the majority rely on VA using a variety of questionnaires.

**Fig. 2:**
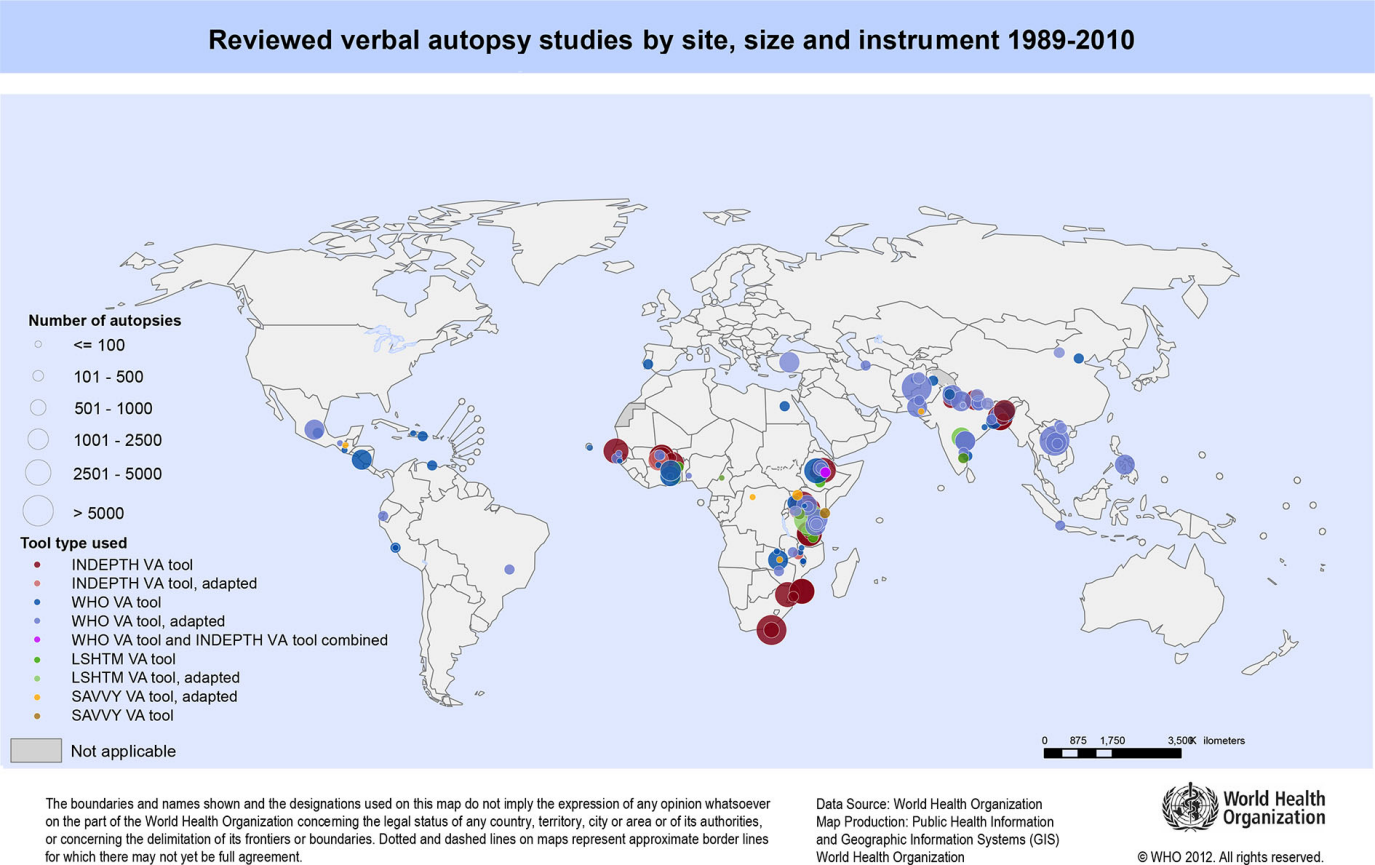
Global distribution of verbal autopsy studies.

Table 1 and Fig. 2 show that the majority of reviewed studies had sites in Africa (54.5%) and Asia (40.2%), while some were conducted in Central and South America (8.9%). The majority of studies using the WHO (61.3%), INDEPTH (64.7%) or adapted versions of the INDEPTH instruments (100%) were in Africa; studies using the WHO (32.3%) or INDEPTH (35.3%) instruments also had sites in Asia.

**Fig. 3:**
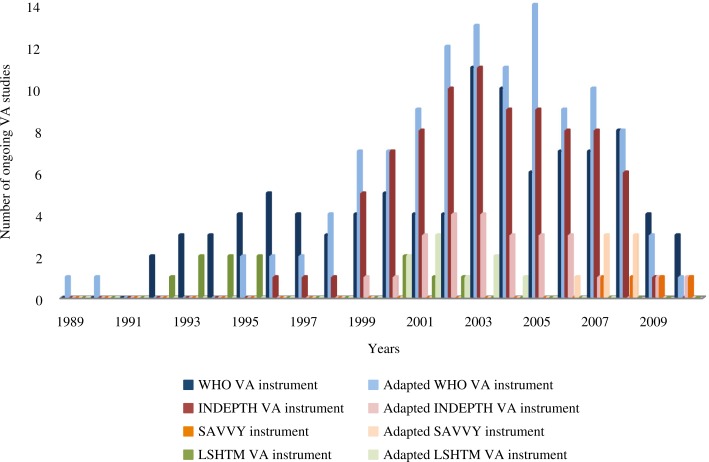
Use of different VA instruments over time.

Use over time for each VA instrument is shown in Fig. 3. Publications using the WHO and INDEPTH VA instruments (and adaptations) increased around 1999, peaking between 2003 and 2005. There have been a limited number of published studies using other instruments. Since the publication of the WHO VA standards in 2007, 17 studies have been conducted which used the WHO VA instrument and adaptations (12/17); the INDEPTH instrument and adaptations (2/17); and the SAVVY instrument and adaptations (3/17). While these figures show that the majority of studies since 2007 have used the WHO VA instrument and adaptations, it is difficult to assess the level of uptake of the WHO VA standards, as trends in more recent data collection years may be difficult to interpret due to delays in publication of results, particularly given delays in PCVA interpretation in some sites.

Age groups were reported by 110 studies. For comparisons, age groups were categorized non-exclusively as: stillbirths; under 4 weeks; 4 weeks to 5 years; under 15 years; 15 years and above; maternal deaths; and all age groups (Table 2). VA instruments have mostly been used for 15 years and above (26.4%) and for all age groups (22.7%). Deaths in children under 5 years old (18.2%) and neonates (18.2%) have also been widely studied.

**Table 2:** Age groups studied by type of VA instrument (*n*=110)^a^

	Stillbirths	Under 4 weeks	Under 5 years	Under 15 years	Aged 15 years and above	Maternal deaths	All age groups
WHO VA instrument	13.3% (4/30)	20.0% (6/30)	30.0% (9/30)	3.3% (1/30)	13.3% (7/30)	10.0% (3/30)	10.0% (3/30)
Adapted WHO VA instrument	7.3% (3/41)	24.4% (10/41)	17.1% (7/41)	0% (0/41)	17.1% (7/41)	17.1% (7/41)	26.8% (11/41)
INDEPTH VA instrument	5.9% (1/17)	11.8% (2/17)	11.8% (2/17)	11.8% (2/17)	17.6% (3/17)	0% (0/17)	47.1% (8/17)
Adapted INDEPTH VA instrument	0% (0/9)	0% (0/9)	22.2% (2/9)	33.3% (3/9)	33.3% (3/9)	0% (0/9)	11.1% (1/9)
SAVVY instrument	0% (0/1)	0% (0/1)	0% (0/1)	0% (0/1)	100.0% (1/1)	0% (0/0)	0% (0/0)
Adapted SAVVY instrument	33.3% (1/3)	66.7% (2/3)	0% (0/3)	0% (0/3)	33.3% (1/3)	0% (0/3)	0% (0/3)
LSHTM VA instrument	0% (0/5)	0% (0/5)	0% (0/5)	0% (0/5)	100.0% (5/5)	0% (0/5)	0% (0/5)
Adapted LSHTM VA instrument	0% (0/4)	0% (0/4)	0% (0/4)	0% (0/4)	50.0% (2/4)	0% (0/4)	50.0% (2/4)
Total	8.2% (9/110)	18.2% (20/110)	18.2% (20/110)	5.5% (6/110)	26.4% (29/110)	9.1% (10/110)	22.7% (25/110)

a Percentages do not add up to 100% as some studies determined CoD in more than one age group.

The most common interpretation method (more than one was used in some studies) was the PCVA (82.9%), followed by probabilistic methods (11.7%), and algorithms (10.8%) (2). Of probabilistic methods, InterVA was most used (61.5%). Only one study used ANN, Random Forest, SSP, Tariff, or King and Lu methods to ascertain CoD.

Validity studies for VA procedures are fraught with difficulties since there is no widely available gold standard, particularly for the majority of LMICs deaths not occurring in health facilities (13). The validity of VA is typically assessed by comparing hospital medical records as gold standard diagnoses for CoD, as well as by making between-method comparisons (e.g. between PCVA and CCVA). The validity of the overall VA process is influenced by the design and content of the questionnaires, field procedures, data interpretation methods, actual CoD patterns, and characteristics of the deceased (14).

Of the 125 studies reviewed, 26 assessed performance of VA procedures in certifying CoD (studies using the same VA dataset but different CoD interpretation methods and/or assessing different validation parameters were included in the review and counted as individual studies) (Table 3 and 4). Apart from adapted versions of the LSHTM VA instrument, all other types of VA instruments have been validated at least once by these studies. The majority of studies validating VA procedures have used the WHO VA instrument (6/26) and their adapted versions (10/26). A similar review by Chandramohan et al. in 1994 identified almost no information on the validity of VA for adult deaths (7). Our review identified that this trend has shifted with most of the 26 studies having assessed the performance for all CoD (21/26), in adults (10/26) and in all age groups (10/26). These studies used a variety of measures, including: sensitivity (14/26); specificity (14/26); positive predictive value (PPV) (8/26); negative predictive value (NPV) (4/26); cause-specific fractions (CSF) (1/26); concordance between CSMF estimated by VA and CSMF from the validation data (10/26); areas under the receiver operator characteristic (ROC) curve (3/26); kappa statistics (7/26); cause-specific and average chance-corrected concordance (5/26); CSMF accuracy (6/26); and cause-specific concordance correlation coefficients of estimated CSMFs compared to true CSMFs (6/26). The ability of these studies to adequately validate VA has often been limited by small sample sizes, affecting reliability of measures such as sensitivity and specificity, and the absence of certain causes from hospital data. Most studies (20/26) relied on hospital CoD data as the standard measure of validity. Exceptions included comparison studies using the InterVA method (7/26), where in five studies the reliability of VA procedure was assessed by the concordance of CSMFs estimated by InterVA and PCVA. The review found two studies validating the performance of InterVA against hospital CoD data (8, 33).

**Table 3:** Main characteristics of reviewed VA validation studies (*n*=26)

Number of validation studies	Measures of validity											Validated against hospital CoD data

	Sensitivity	Specificity	PPV	NPV	CSF	Concordance between VA CSMF and CSMF from validation data	ROC curve	Kappa statistics	Cause-specific and average chance-corrected concordance	CSMF accuracy	Cause-specific concordance correlation coefficients of estimated CSMFs compared to true CSMFs	
WHO VA instrument (*n*=6)	6/6	6/6	2/6	2/6	1/6	1/6	1/6	0/6	0/6	0/6	0/6	6/6
Adapted WHO VA instrument (*n*=10)	3/10	2/10	2/10	1/10	0/10	2/10	0/10	2/10	5/10	6/10	6/10	9/10
INDEPTH VA instrument (*n*=3)	0/3	0/3	0/3	0/3	0/3	2/3	0/3	2/3	0/3	0/3	0/3	0/3
Adapted INDEPTH VA instrument (*n*=1)	1/1	1/1	1/1	0/1	0/1	1/1	1/1	1/1	0/1	0/1	0/1	0/1
SAVVY instrument (*n*=1)	1/1	1/1	1/1	1/1	0/1	1/1	1/1	1/1	0/1	0/1	0/1	1/1
Adapted SAVVY instrument (*n*=1)	0/1	1/1	1/1	0/1	0/1	0/1	0/1	0/1	0/1	0/1	0/1	0/1
LSHTM VA instrument (*n*=4)	3/4	3/4	1/4	0/4	0/4	3/4	0/4	1/4	0/4	0/4	0/4	4/4
Total (*n*=26)	14/26	14/26	8/26	4/26	1/26	10/26	3/26	7/26	5/26	6/26	6/26	20/26

**Table 4:** List of reviewed VA validation studies (*n*=26)

Instrument and source	VA interpretation method	Number of deaths certified	CoD studied	Age groups studied	Validity and reliability parameters
WHO VA instrument (15)	Physician review	225	All causes	Stillbirths	Sensitivity, Specificity, PPV, NPV, ROC curves
WHO VA instrument (16)	Algorithms	1115	Diarrhoea and pneumonia	Children under 5 years	Sensitivity, Specificity, CSMF
WHO VA instrument (17)	Physician review	719	All causes	Children under 5 years	Sensitivity, Specificity, PPV, Difference between CSMF estimated by VA and true CSMF in validation data
WHO VA instrument (18)	Physician review	763	Stroke	Adults	Sensitivity, Specificity
WHO VA instrument (19)	Physician review	1 251	All causes	Stillbirths and neonates	Sensitivity, Specificity
WHO VA instrument (20)	Physician review	36	Selected childhood illnesses	Children under 12 years old	Sensitivity, Specificity, PPV, NPV
Adapted WHO VA instrument (21)	Physician review	255	All causes	All age groups	Sensitivity, Specificity
Adapted WHO VA instrument (22)	Physician review	219	All causes	Adults	Sensitivity, Specificity, PPV, NPV, Kappa statistics
Adapted WHO VA instrument (23)	Physician review and InterVA	734	All causes	Stillbirths and neonates	Concordance of CSMFs estimated by InterVA and physician review, Level of agreement between InterVA and physician assigned CoD using Kappa statistics
Adapted WHO VA instrument (24)	Physician review	9 817	All causes	All age groups	Sensitivity, PPV, Concordance of CSMFs estimated by physician review and medical record diagnoses
Adapted WHO VA instrument (8)	InterVA, physician review and SP method	12 542	All causes	All age groups	Average of cause-specific chance-corrected concordance, CSMF accuracy, relationship between estimated CSMFs and true CSMFs
Adapted WHO VA instrument (25)	King Lu method and physician review	12 542	All causes	All age groups	CSMFs accuracy, relationship between estimated CSMFs and true CSMFs
Adapted WHO VA instrument (26)	Physician review	12 542	All causes	All age groups	Average of cause-specific chance-corrected concordance, CSMFs accuracy, Relationship between estimated CSMFs and true CSMFs
Adapted WHO VA instrument (27)	Tariff method and physician review	12 542	All causes	All age groups	Average of cause-specific chance-corrected concordance, CSMFs accuracy, Relationship between estimated CSMFs and true CSMFs
Adapted WHO VA instrument (28)	SSP method and physician review	12 542	All causes	All age groups	Average of cause-specific chance-corrected concordance, CSMFs accuracy, Relationship between estimated CSMFs and true CSMFs
Adapted WHO VA instrument (6)	Random Forests method and physician review	12 542	All causes	All age groups	Average of cause-specific chance-corrected concordance, CSMFs accuracy, Relationship between estimated CSMFs and true CSMFs
INDEPTH VA instrument (29)	Physician review and InterVA	1 823	All causes	Children under 5 years and adults	Level of agreement between InterVA and physician assigned CoD using Kappa statistics, Concordance of CSMFs estimated by InterVA and physician review
INDEPTH VA instrument (30)	Physician review and InterVA	10 267	All causes	All age groups	Level of agreement between InterVA and physician assigned CoD using Kappa statistics
INDEPTH VA instrument (31)	Physician review and InterVA	289	All causes	All age groups	Concordance of CSMFs between InterVA and physician review
Adapted INDEPTH VA instrument (32)	InterVA	193	HIV/AIDS	Adults	Sensitivity, Specificity, PPV, Concordance of CSMFs between InterVA and the reference standard, Level of agreement between InterVA and reference standard CoD using Kappa statistics, ROC curves
SAVVY instrument (33)	Physician review and InterVA	145	All causes	Adults	Sensitivity, Specificity, PPV, NPV, ROC curves, Level of agreement between InterVA, physician review and hospital CoD using Kappa statistics, Concordance of CSMFs between InterVA, physician review and hospital CoD
Adapted SAVVY instrument (34)	Physician review	264	HIV/AIDS	Adults	Specificity, PPV
LSHTM VA instrument (35)	Physician review and algorithms	615	All causes	Adults	Sensitivity, Specificity, Concordance of CSMF obtained using the data-derived algorithms with the CSMF obtained using physician review
LSHTM VA instrument (36)	Physician review and expert algorithms	796	All causes	Adults	Sensitivity, Specificity, PPV, Concordance of CSMFs between physician review, algorithms and hospital CoD
LSHTM VA instrument (37)	Physician review, expert algorithms and data-derived algorithms	796	All causes	Adults	Sensitivity, Specificity, Concordance of CSMFs between physician review, algorithms and hospital CoD
LSHTM VA instrument (38)	Data-derived algorithms	40	All causes	Adults	Kappa statistics

Our review of VA studies published up to 2012 highlights variability in the selection, development, and use of VA instruments, as well as in methods of assessment. The review established that there are many adaptations of standard VA instruments. Although instruments may need to be adapted to local contexts, the extent of modifications was not reported by studies and their impact on VA performance and accuracy are not known. The review was hindered by an absence of information on the VA instrument used by a substantial number of studies. The lack of systematic detailed information on methods used undermines the value of experience sharing on use of VA instruments and limits a more accurate understanding of the use of the different instruments and uptake of VA guidelines. Some reports on using VA may have been missed if written in other languages or as yet unpublished.

## Simplification of VA standards: the 2012 WHO VA instrument

In December 2011, following the above review process, consensus over a simplified VA instrument was reached among 37 experts from 15 countries in a meeting organized by WHO in collaboration with the University of Queensland, the Health Metrics Network and INDEPTH. The meeting was followed by a 2-day workshop during which the outcomes of the discussions were consolidated. Participants included key stakeholders, researchers, and those who work routinely with VA instruments. The 2012 WHO VA instrument comprises a total of 221 CoD-related indicators to certify 62 CoD. The instrument is designed primarily for electronic data capture, and WHO data collection software will facilitate this on generic mobile devices. CoD interpretation software also allows assessment without physicians, reducing cost and time lag in VA interpretation, and enhancing comparability across different settings and over time. For those wanting to use paper capture and PCVA, simplified sample questionnaires have been developed for three age groups: under 4 weeks; 4 weeks to 14 years; and 15 years and over, which are available with all other aspects of the 2012 WHO VA instrument at www.who.int/healthinfo/statistics/verbalautopsystandards

As determined by extensive skip patterns, the maximum number of questions to be asked for any death ranges from 104 for a neonatal death to 130 for a maternal death (Table 5). Although users may need to add locally relevant questions, the instrument as defined here should be regarded as the core.

**Table 5:** Pattern of indicators by age group

	Number of indicators								

Age group	CoD-related								
				
Skip level	First	Second	Third	Fourth	Total	Personal	Respondent	Context	Total
15+ years	56	37	27	10	130	26	3	10	169
4 weeks–14 years	34	35	22	10	101	26	3	10	140
Under 28 days	44	35	15	10	104	26	3	10	143
Total	93	87	31	10	221				

## Simplified list of CoD

To develop a VA instrument appropriate for strengthening countries’ CRVS systems, we simplified the WHO VA standards; this commenced with generating a shorter list of CoD. Three main criteria characterized essential CoD:

1. Importance: most frequent CoD of global public health relevance (e.g. acute respiratory infections);
2. Diagnostic Feasibility: CoD associated with recognizable symptoms ascertainable by VA (e.g. HIV/AIDS); and
3. Potential for intervention: CoD can be addressed by public health interventions (e.g. diarrhoeal diseases).

Comparison of the results of most widely used and validated VA instruments and interpretation approaches including PCVA, InterVA, and Population Health Metrics Research Consortium (PHMRC) methods, enabled the identification of a core group of CoD that can be certified by VA. This core group of CoD was mapped against the 31 causes reported in the 2004 Global Burden of Disease (GBD) study to ascertain the public health importance of individual causes. Finally, consensus on the simplified list of CoD was reached in the meeting with VA experts, based on their experience and available evidence.

In the 2007 WHO VA standards, there were 106 possible CoD to be assigned by physicians, while InterVA-3 and InterVA-M assigned 48 causes and the PHMRC VA instrument reached 51 (5, 31, 39). To facilitate comparison, some CoD from the WHO VA standards were re-categorized, creating a set of mutually exclusive, collectively exhaustive CoD categories. Table 6 displays the results from the review and correlation of CoD between the VA instruments and the GBD.

**Table 6:** Correspondence of CoD between the 2007 WHO VA standards, InterVA and PHMRC VA instruments, the 2004 GBD, and their reported percentage in 125 reviewed VA studies

2007 WHO VA standards	2004 GBD	InterVA	PHMRC VA instrument	% Reported in various studies
Infectious and parasitic diseases				
Sepsis		•	•	10.4
Acute respiratory infection, including pneumonia	•	•	•	37.6
HIV/AIDS related death	•	•	•	36.8
Intestinal infectious diseases	•	•	•	40.8
Malaria	•	•	•	33.6
Measles	•	•	•	10.4
Meningitis	•	•	•	30.4
Tetanus		•		4.8
Pulmonary tuberculosis	•	•	•	35.2
Typhoid and Paratyphoid				0.8
Pertussis	•			2.4
Leishmaniasis				0
Viral hepatitis				6.4
Arthropod-borne viral fevers and viral haemorrhagic fevers			•	4.0
Other infectious disease, unspecified		•	•	21.6
Neoplasms				
Oral neoplasms	•			4.0
Digestive neoplasms	•		•	12.0
Malignant neoplasm of rectum and anus	•		•	4.8
Respiratory neoplasms	•		•	8.0
Breast neoplasms	•		•	4.8
Reproductive neoplasms	•		•	10.4
Melanoma of skin				0
Neoplasm of lymphoid, haematopoietic and related tissue				0.8
Other and unspecified neoplasms		•	•	20.0
Nutritional and endocrine disorders				
Severe anaemia				9.6
Severe malnutrition	•	•		16.0
Diabetes mellitus	•	•	•	14.4
Other and unspecified nutritional and endocrine disorders				1.6
Diseases of circulatory system				
Acute cardiac disease	•	•	•	16.0
Sickle cell		•		0.8
Cerebrovascular disease	•	•	•	22.4
Other and unspecified cardiac disease			•	44.0
Respiratory disorders				
Chronic obstructive pulmonary disease (COPD)			•	6.4
Asthma			•	4.8
Other and unspecified respiratory disease		•		20.8
Gastrointestinal diseases				
Acute abdominal condition				6.4
Chronic liver disorder	•	•	•	16.0
Other and unspecified digestive disease		•	•	13.6
Renal disorders				
Renal failure	•	•	•	14.4
Other and unspecified disorders of kidney and ureter				2.4
Mental and nervous system disorders				
Mental disorder				2.4
Disease of nervous system		•		3.2
Epilepsy/Acute seizures			•	4.8
Pregnancy-, childbirth and puerperium-related disorders				
Ectopic pregnancy		•		1.6
Abortion-related death		•		4.0
Pregnancy-induced hypertension		•		9.6
Obstetric haemorrhage		•		12.8
Obstructed labour		•		5.6
Pregnancy-related sepsis		•		8.8
Anaemia of pregnancy		•		1.6
Ruptured uterus		•		3.2
Other and unspecified maternal cause		•	•	20.8
Perinatal causes of death				
Prematurity	•	•	•	35.2
Perinatal asphyxia	•	•	•	29.6
Neonatal pneumonia	•		•	8.8
Neonatal sepsis	•		•	20.8
Neonatal tetanus	•			10.4
Congenital malformation	•	•	•	27.2
Other diseases related to the perinatal period, unspecified				12.0
Stillbirth			•	8.0
External causes				
Road traffic accident	•	•	•	9.6
Other transport accident	•	•		7.2
Accidental fall		•	•	6.4
Accidental drowning and submersion		•	•	9.6
Accidental exposure to smoke, fire and flames	•	•	•	7.2
Contact with venomous animals and plants		•	•	3.2
Accidental poisoning and exposure to noxious substance		•	•	5.6
Intentional self-harm	•	•	•	15.2
Assault, homicide, war	•	•	•	14.3
Exposure to force of nature				0
Lack of food and/or water				0
Legal intervention				0
Accident, unspecified		•		14.4
Other and unspecified external cause		•	•	25.6

In the review of 125 studies covering 199,158 deaths described above, we collated evidence on CoD certified by VA and reported in studies to illustrate the range of CoD that were observed and certifiable by VA. The top 10 CoD reported were: ‘other and unspecified cardiac disease’ (44%); ‘intestinal infectious diseases’ (40.8%); ‘acute respiratory infections, including pneumonia’ (37.6%); ‘HIV/AIDS-related death’ (36.8%); ‘pulmonary tuberculosis’ (35.2%); ‘prematurity’ (35.2%); ‘malaria’ (33.6%); ‘perinatal asphyxia’ (29.6%); ‘congenital malformations’ (27.2%); and ‘Other and unspecified external cause of death’ (25.6%). In contrast, the 10 CoD certified and reported least frequently were: ‘typhoid and paratyphoid’ (0.8%); ‘neoplasm of lymphoid, haematopoietic and related tissue’ (0.8%); ‘sickle cell’ (0.8%); ‘ectopic pregnancy’ (1.6%); ‘anaemia of pregnancy’ (1.6%); ‘other and unspecified nutritional and endocrine disorders’ (1.6%); ‘other and unspecified disorders of kidney and ureter’ (2.4%); ‘mental disorder’ (2.4%); ‘pertussis’ (2.4%); and ‘disease of nervous system’ (3.2%). The CoD ‘Leishmaniasis’, ‘melanoma of skin’, ‘exposure to force of nature’, ‘lack of food and/or water’, and ‘legal intervention’ have not been certified by VA in any of the reviewed studies.

Elimination of CoD was based on low frequency reported by VA studies, not being included in the other VA instruments, and on experts’ judgment about their importance, feasibility and intervention potential. As a result, 27 CoD from the 2007 WHO VA standards were subsumed into residual categories, including ‘typhoid and paratyphoid’, ‘leishmaniasis’, ‘melanoma of skin’, ‘lack of food and/or water’ and ‘legal intervention’ (Table 7).

**Table 7:** CoD removed from CoD list of the 2007 WHO VA standard and subsumed into other categories in 2012 WHO standard (*n*=27)

2007 WHO VA standard cause	Subsumed into 2012 WHO VA cause
Other digestive disease	VAs-98
Typhoid and paratyphoid	VAs-01.99
Viral hepatitis	VAs-01.99
Leishmaniasis	VAs-01.99
Malignant melanoma of skin	VAs-02.99
Malignant neoplasm of lymphoid, haematopoietic and related tissue	VAs-02.99
Other specified neoplasms	VAs-02.99
Other specified endocrine disorders	VAs-98
Endocrine disorders, unspecified	VAs-98
Other specified diseases of the respiratory system	VAs-98
Respiratory disorder, unspecified	VAs-98
Respiratory failure, not elsewhere classified	VAs-98
Other diseases of intestine	VAs-98
Disease of intestine, unspecified	VAs-98
Specified mental disorders	VAs-98
Mental disorders, unspecified	VAs-98
Other specified disorders of the nervous system	VAs-98
Nervous system disorders, not otherwise classified	VAs-98
Alzheimer's disease	VAs-98
Other specified direct maternal causes	VAs-09.99
Congenital viral diseases	VAs-01.99
Congenital malformations of the nervous system	VAs-10.06
Other specified disorders related to perinatal period	VAs-10.99
Lack of food and/or water	VAs-12.99
Legal intervention	VAs-12.99
Accident, unspecified	VAs-12.99
Other specified event, undetermined intent	VAs-12.99

The inclusion of the majority of CoD in the simplified CoD list was based on the consistency between CoD from WHO VA standards against InterVA and PHMRC VA, GBD estimates and coverage in VA studies. All causes included in the GBD and the top 10 most certified CoD reported were retained. During expert meetings, the CoD ‘other and unspecified non-communicable disease’, ‘sepsis’, ‘anaemia of pregnancy’ and ‘ruptured uterus’ were added to the list. Although not in the GBD or most commonly certified CoD, they were considered feasible for VA certification, provide key information to CRVS, contribute significant mortality burdens and are responsive to interventions. Further modifications included grouping related CoD not having readily distinguishable symptoms into broader categories. For example, ‘malignant neoplasm of cervix’ and ‘malignant neoplasm of uterus’ were combined into ‘female reproductive neoplasms’. Overall, the simplification process led to a 41.5% reduction in CoD compared with the WHO VA standards CoD list, resulting in 60 CoD. A further two categories were added for fresh and macerated stillbirths, despite not strictly considered as CoD, because of their importance in some settings. Table 8 presents the simplified VA CoD list, structuring the causes into groupings consistent with ICD-10 and showing in the last column how all ICD-10 codes map onto the 62 CoD.

**Table 8:** Simplified CoD list for 2012 WHO VA with corresponding ICD-10 codes

2012 verbal autopsy code	Verbal autopsy title	ICD-10 code (to ICD)	ICD-10 codes (from ICD)
VAs-01	Infectious and parasitic diseases		
VAs-01.01	Sepsis	A41	A40–A41
VAs-01.02	Acute respiratory infection, including pneumonia	J22; J18	J00–J22
VAs-01.03	HIV/AIDS related death	B24	B20–B24
VAs-01.04	Diarrheal diseases	A09	A00–A09
VAs-01.05	Malaria	B54	B50–B54
VAs-01.06	Measles	B05	B05
VAs-01.07	Meningitis and encephalitis	G03; G04	A39; G00–G05
VAs-01.08	Tetanus	A35 (obstetric A34)	A33–A35
VAs-01.09	Pulmonary tuberculosis	A16	A15–A16
VAs-01.10	Pertussis	A37	A37
VAs-01.11	Haemorrhagic fever	A99	A90–A99
VAs-01.99	Other and unspecified infectious disease	B99	A20–A38; A42–A89; B00–B19; B25–B49; B55–B99
	Non-communicable diseases		
VAs-98	Other and unspecified non-communicable disease	R99	D55–D89; E00–E07; E15–E35; E50–E90; F00–F99; G10–G37; G50–G99; H00–H95; J30–J39; J47–J99; K00–K31; K40–K93; L00–L99; M00–M99; N00–N16; N20–N99; R00–R69
VAs-02			Neoplasms
VAs-02.01	Oral neoplasms	C06	C00–C06
VAs-02.02	Digestive neoplasms	C26	C15–C26
VAs-02.03	Respiratory neoplasms	C39	C30–C39
VAs-02.04	Breast neoplasms	C50	C50
VAs-02.05	Female reproductive neoplasms	C57	C51–C58
VAs-02.06	Male reproductive neoplasms	C63	C60–C63
VAs-02.99	Other and unspecified neoplasms	C80	C07–C14; C40–C49; C60–D48
VAs-03	Nutritional and endocrine disorders		
VAs-03.01	Severe anaemia	D64	D50–D64
VAs-03.02	Severe malnutrition	E46	E40–E46
VAs-03.03	Diabetes mellitus	E14	E10–E14
VAs-04	Diseases of the circulatory system		
VAs-04.01	Acute cardiac disease	I24 (acute ischemic)	I20–I25
VAs-04.02	Stroke	I64	I60–I69
VAs-04.03	Sickle cell with crisis	D57	D57
VAs-04.99	Other and unspecified cardiac disease	I99	I10–I15; I26–I52; I70–I99
VAs-05	Respiratory disorders		
VAs-05.01	Chronic obstructive pulmonary disease (COPD)	J44	J40–J44
VAs-05.02	Asthma	J45 (J46)	J45–J46
VAs-06	Gastrointestinal disorders		
VAs-06.01	Acute abdomen	R10	R10
VAs-06.02	Liver cirrhosis	K74	K70–K76
VAs-07			Renal disorders
VAs-07.01	Renal failure	N19	N17–N19
VAs-08	Mental and nervous system disorders		
VAs-08.01	Epilepsy	G40	G40–G41
VAs-09	Pregnancy-, childbirth and puerperium-related disorders		
VAs-09.01	Ectopic pregnancy	O00	O00
VAs-09.02	Abortion-related death	O06	O03–O08
VAs-09.03	Pregnancy-induced hypertension	O13 (or O15 for eclampsia)	O10–O16
VAs-09.04	Obstetric haemorrhage	O46 (antepartum) O72 (postpartum)	O46; O67; O72
VAs-09.05	Obstructed labour	O66	O63–O66
VAs-09.06	Pregnancy-related sepsis	O75.3 (antepartum) O85 (postpartum)	O85; O75.3
VAs-09.07	Anaemia of pregnancy	O99	O99.0
VAs-09.08	Ruptured uterus	O71	O71
VAs-09.99	Other and unspecified maternal cause	O05	O01–O02; O20–O45; O47–O62; O68–O70; O73–O84; O86–O99
VAs-10	Neonatal causes of death		
VAs-10.01	Prematurity	P07	P05–P07
VAs-10.02	Birth asphyxia	P21	P20–P22
VAs-10.03	Neonatal pneumonia	P23	P23–P25
VAs-10.04	Neonatal sepsis	P63	P36
VAs-10.05	Neonatal tetanus	A33	A33
VAs-10.06	Congenital malformation	Q89	Q00–Q99
VAs-10.99	Other and unspecified perinatal cause of death	P96	P00–P04; P08–P15; P26–P35; P37–P94; P96
VAs-11	Stillbirths		
VAs-11.01	Fresh stillbirth	P95	P95
VAs-11.02	Macerated stillbirth	P95	P95
VAs-12	External causes of death		
VAs-12.01	Road traffic accident	V89	V01–V89
VAs-12.02	Other transport accident	V99	V90–V99
VAs-12.03	Accidental fall	W19	W00–W19
VAs-12.04	Accidental drowning and submersion	W74	W65–W74
VAs-12.05	Accidental exposure to smoke, fire and flames	X09	X00–X19
VAs-12.06	Contact with venomous animals and plants	X29	X20–X29
VAs-12.07	Accidental poisoning and exposure to noxious substance	X49	X40–X49
VAs-12.08	Intentional self-harm	X84	X60–X84
VAs-12.09	Assault	Y09	X85–Y09
VAs-12.10	Exposure to force of nature	X39	X30–X39
VAs-12.99	Other and unspecified external cause of death	X59	S00–T99; W20–W64; W75–W99; X50–X59; Y10–Y98
VA-99	Cause of death unknown	R99	R99

## VA questionnaires and indicators

VA questionnaires ask specific questions about signs, symptoms, complaints, or contextual factors that will lead to determining the most probable CoD. Such information that indicates the possibility of specific causes is inclusively termed as ‘indicators’. The review aimed to collate evidence from field experience on: (i) specific modifications made to VA questionnaires and their rationales; (ii) utility of specific indicators for CoD ascertainment; and (iii) identification of most and least specific indicators for reaching diagnoses. From the 125 studies reviewed, contact was attempted with 45 randomly selected authors (one per study, unless referred to another), and established with 27. Limited feedback was gathered on specific indicators, as most researchers were not able to report on specific modifications made to the VA instruments, and they found it challenging to give feedback on the utility, value and specificity of individual questionnaire indicators. The following alterations to standard instruments were reported:

1. Structural rearrangement of order and categorization of questionnaire modules and changes in targeted age groups;
2. Attempts to shorten the VA questionnaires by removal and modification of questions related to the duration of signs and symptoms; and
3. Addition of disease-specific questions for local conditions and research needs.

Overall, users considered the 2007 WHO VA standards too long and time-consuming, expressing a desire for shorter and more practical instruments. This process of simplification was started by drafting diagnostic criteria for each CoD by listing symptoms indicated in the Oxford Text Book of Medicine (40). Subsequently, experts identified essential indicators for differentiating CoD, and inclusion/exclusion was based on likely recognition, recollection, and reporting in VA interviews. Evidence of indicators’ utility was gathered by correlating indicators from WHO VA standards, InterVA, and PHMRC VA procedures. Furthermore, the simplification of the WHO VA standards indicators was informed by a progressive item reduction process based on the Tariff method (27). Participating experts from PHMRC had applied the Tariff method to the PHMRC validation dataset and tested the effect of dropping items or sets of items on chance-corrected concordance and CSMF accuracy. These findings comprised one element of the discussion on the evidence base for some CoD. Indicators removed had low specificity and possibly generated answers with low reliability due to recall difficulties. These were mainly sub-indicators on the duration, frequency, and development of signs and symptoms. Other modifications made included the addition of indicators from InterVA and PHMRC VA instruments, the removal of overlapping indicators capturing very similar information, and the inclusion of social context indicators, facilitating use of the instrument in non-enumerated populations. Overall, 164 indicators were retained from the 2007 WHO VA standard, 57 new indicators introduced and 244/408 indicators from the 2007 WHO VA standard excluded. Review by expert groups – for relevance to the list of causes, reliability, and feasibility – and comparison with machine assessment analysis led to a reduction of 45.8% in number of CoD-related indicators in relation to the WHO VA standards, resulting in a total of 221 indicators (of which various subsets apply to particular population sub-groups).

## Application of the 2012 WHO VA instrument to facilitate routine surveillance

The need for consensus on simplified technical standards and guidelines for VA, together with their widespread endorsement and adoption, has become urgent. The systematic use of the 2012 WHO VA instrument will strengthen countries’ CRVS systems. In the past decade, methodological developments in automated methods for VA assessment have created a shift away from limited individual-level and clinical paradigms towards population-based epidemiological and public health thinking. To facilitate application in routine surveillance systems, the new simplified VA instrument was specifically developed for automated ascertainment of CoD. At present, the InterVA-4 model, as previously described (41), is the only available automated interpretation tool fully aligned with the 2012 WHO VA instrument. A simple, automatically interpreted VA process will lead to increased coverage of operational and representative CRVS systems. Shorter and simpler interviews not needing physicians for CoD interpretation will facilitate collection of adequate data for CRVS systems. CCVA brings efficiency and consistency by providing a standardized interpretation of VA. The new 2012 WHO VA instrument will be piloted, modified, and integrated into national health information systems.

## Footnotes

1 INDEPTH (www.indepth-network.org) is a network of member centres that conduct longitudinal health and demographic evaluation of populations in LMICs. INDEPTH has built a network of 44 health and demographic surveillance systems (HDSS) across 20 countries in Africa, Asia, and Oceania. The network strengthens capacity of HDSS centres, and mounts multicentre research to guide health priorities and policies in LMICs, based on up-to-date empirical scientific evidence. The network uses VA as a method for determining CoD.

2 SAVVY, proposed by MEASURE Evaluation and the International Programs Center, U.S. Census Bureau, is a system to generate reliable information on mortality levels and CoD at the national level. The SAVVY resource library is a series of best practice manuals and methods for improving the quality of vital statistics where high coverage of civil registration and good CoD data are not available. A SAVVY system collects mortality data from a number of sites throughout a country using multistage probability sampling. SAVVY Methods include determination of CoD with VA.
